# Clarifying the Temporal Dynamics of the Circadian Clock and Flowering Gene Network Using Overexpression and Targeted Mutagenesis of Soybean *EARLY FLOWERING 3-1* ( *GmELF3-1* )

**DOI:** 10.17912/micropub.biology.000935

**Published:** 2023-10-15

**Authors:** Michelle Alcantara, Hira Iftikhar, Kimberly Kagan, Dianna Dzheyranyan, Pedram Abbasi, Alejandra Alamilla, Nicole Ayala, Trixy Baca, Vanessa Benoit, Natalia Clausen, Caroline Coto, Celia Guerrero, Erik Hernandez Catalan, Sierra Hurtado, Angela Lopez, Jacqueline Lopez, Nicholas Majarian, Noah Mesfin, Avetis Mishegyan, Goharik Mkrtchyan, Amy Ordonez, Arthur Pachanyan, Tanya Pelayo, Alondra Rosas, Kylee Rowsey, Elina Sharma, Sanjiti Sharma, Shauna Van Grinsven, Yoshie Hanzawa

**Affiliations:** 1 Department of Biology, California State University Northridge; 2 Department of Biology, BIOL 481L Plant Physiology, California State University Northridge

## Abstract

With progressing climate fluctuations, an understanding of the molecular mechanisms of crop plants that regulate their flowering responses to environments is crucial. To achieve this goal, we aimed at clarifying the gene regulatory networks among the circadian clock and flowering genes in soybean ( *Glycine max* ). Based on our network inference approach *,* we hypothesize that *GmELF3-1* , one of the Evening Complex (EC) gene homologs in soybean’s circadian clock, may have an integrative role in transcriptional regulation of the circadian clock and flowering gene network. In this study, we verify GmELF3-1 *’* s regulatory roles in its potential downstream genes by modulating the activity of GmELF3-1 using overexpression and CRISPR-Cas9 in soybean protoplasts. Our results indicate that GmELF3-1 may control the expression of the *PRR* genes in the circadian clock and the flowering gene *GmCOL1a* .

**Figure 1. The regulatory effect of GmELF3-1 on inferred downstream genes with overexpression and CRISPR-Cas9 mutagenesis of GmELF3-1 in soybean protoplasts.**
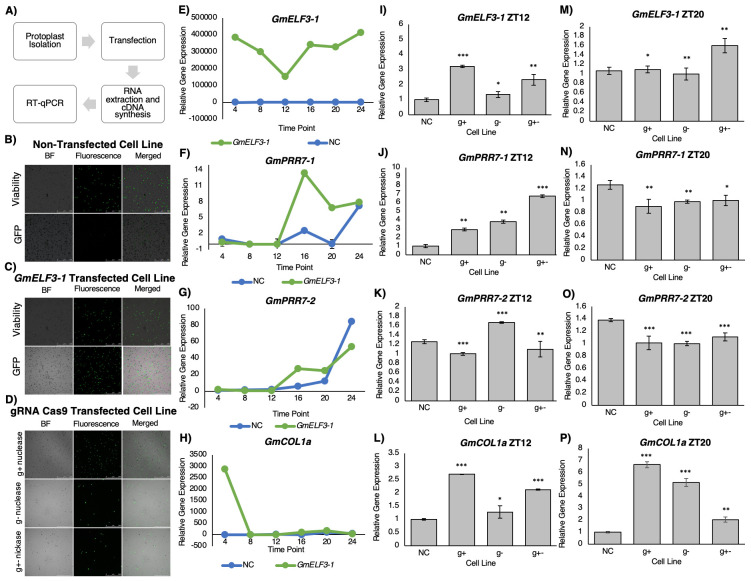
**A)** Flowchart of experimental steps used in this study. **B-D)** Representative images of non-transfected (B), *GFP-GmELF3-1* transfected (C), and CRISPR-Cas9 transfected (D) protoplasts in bright field (BF) and fluorescent light at a wavelength of 523nm (514-527nm range) showing cell viability and GFP expression. To determine cell viability, protoplasts were stained with FDA at the time of harvest. **E-H)** Relative expression of *GmELF3-1* (E) and inferred *GmELF3-1* target genes: *GmPRR7-1* (F), *GmPRR7-2* (G), and *GmCOL1b* (H) in non-transfected negative control protoplasts (blue) and *GFP-GmCOL1b* transfected protoplasts (green) at Zeitgeber time points ZT4-ZT24 by RT-qPCR in two biological samples with three technical replications. **I-P)** Relative expression of *GmELF3-1* (I and M), *GmPRR7-1* (J and N), *GmPRR7-2* (K and O), and *GmCOL1b* (L and P) in negative control protoplasts (NC) and RNP transfected protoplasts at ZT12 (I-L) and at ZT20 (M-P) after over 48 hours incubation by RT-qPCR in two biological samples with three technical replications. The following RNP complexes were used: sgRNA on the positive strand with Cas9 nuclease (g+), sgRNA on the negative strand with Cas9 nuclease (g-), or paired guide RNA with Cas9 nickase (g+-). Normalized expression levels were calculated as 2 ^-∆Ct^ against the housekeeping gene *GmPBB2* as described previously (Livak & Schmittgen, 2001; Wu et al., 2014). Error bars indicate standard error with 2 biological replicates. One-way ANOVA was used for statistical tests. *P<0.05, **P<0.01, ***P<0.001.

## Description

To adapt to temperature and light fluctuations, sessile plants have evolved internal machineries to sense and acclimate to these environmental changes to maintain their homeostatic balance (Creux & Harmer, 2019; McClung, 2019) . Anticipation of external conditions have allowed the development of an internal timing mechanism, the circadian clock, to align their key biological processes to a 24-hour period. In the model plant *Arabidopsis thaliana* (Arabidopsis), the circadian clock is a complex gene regulatory network consisting of multiple intertwined feedback loops of transcriptional repressors that creates daily rhythms and influences a wide range of physiological processes, including flowering transition in plants (Harmon et al. 2018; Creux & Harmer, 2019; Ronald and Davis 2019) . *LATE ELONGATED HYPOCOTYL* ( *LHY* ) and *CIRCADIAN CLOCK ASSOCIATED 1* ( *CCA1* ) genes that encode the closely related MYB family transcription factors (Alabadí, Yanovsky, Más, Harmer, & Kay, 2002; Mizoguchi et al., 2002) are expressed in the morning and repress *PSEUDO-RESPONSE REGULATOR* ( *PRR* ) genes, including *TIMING OF CAB2 EXPRESSION 1* ( *TOC1* ), *PRR5* , *PRR7* , and *PRR9* . These *PRR* genes are expressed in the afternoon and repress *LHY* and *CCA1* genes in turn, creating a feedback loop (Nakamichi et al., 2010; Nakamichi et al., 2012) ). *LHY* and *CCA1* also repress the components of the Evening Complex (EC): the transcriptional repressor *EARLY FLOWERING 3* ( *ELF3* ) *,* the nuclear protein *ELF4,* and the MYB transcription factor *LUX ARRHYTHMO* ( *LUX* ) (Huang & Nusinow, 2016) . The EC genes in turn repress *LHY* , *CCA1* , and multiple *PRR* genes (Chow, Helfer, Nusinow, & Kay, 2012; Ezer et al., 2017; Mizuno et al., 2014) .

Our current knowledge in the molecular basis underlying the circadian clock in crop species is limited. In soybean ( *Glycine max* ), recent studies have reported that soybean’s clock gene homologs affect photoperiodic flowering transition by modulating the *E1* gene, a legume specific flowering repressor (Xia et al., 2012) . The soybean LHY homolog LHY1a directly binds to the promoter of the *E1* gene and inhibits its expression (Lu et al., 2020) , while *GmPRR3* genes repress *LHY/CCA1* homologs, derepressing *E1* expression (Li et al., 2020; Lu et al., 2020) . In addition, components of the evening complex (EC), GmELF3 and GmLUX homologs, are shown to directly inhibit *E1* expression (Bu et al., 2021; Lu et al., 2017) . In Arabidopsis, the EC is known to play a central role in the entrainment of the circadian clock and the coordination of plant growth and environmental signals (Ezer et al., 2017; Huang & Nusinow, 2016) . Similarly, the soybean *ELF3* homolog *GmELF3-1* , the causal gene for the *J* locus that confers a long juvenile trait, is involved in the latitudinal adaptation of soybean (Lu et al., 2017; Yue et al., 2017) . Soybean is a short-day flowering plant, and its yield is critically dependent on the photoperiod in each latitudinal zone. Recessive mutations at *GmELF3-1* caused late flowering and higher yield under short day (SD) conditions via upregulation of *E1* , providing the better adaption in tropic, low-latitude zones such as Brazil, the second largest producer of soybean. Therefore, GmELF3-1 is a crucial component of a regional expansion of soybeans.

In this study, we aimed to gain a better understanding of the regulatory roles of the EC gene *GmELF3-1* in the circadian clock and flowering gene network of soybean. To test the regulatory effects of GmELF3-1 on downstream gene expression, soybean protoplast cells were used as a transient model. Previously, regulatory interactions among 82 soybean’s circadian clock and flowering genes were inferred using in-house time series RNA-seq data and the network inference algorithmic package CausNet (Wu et al., 2019) . Candidate target genes downstream of GmELF3-1 were identified: *GmCOL1a, GmPRR7-1,* and *GmPRR7-2* (Alcantara et al., 2022) . *GmPRR7-1* and *GmPRR7-2* were predicted to be downregulated by GmELF3-1 with strong reliability weights of 0.62 and 0.72, respectively, in the photothermal condition long day (LD) at 25°C. *GmCOL1a* was upregulated by GmELF3-1 with a very strong reliability weight of 1.0, in LD at 16°C.

To verify these inferred regulatory interactions experimentally, the effects of GmELF3-1 in the candidate downstream genes were examined using soybean protoplast cells as a transient model in two ways. In the first approach, *GmELF3-1* was overexpressed in protoplast cells by transfecting them with *GFP-GmELF3-1* fused to the Cauliflower Mosaic Virus (CaMV) 35S promoter **(Table 2)** . By constitutively expressing *GFP-GmELF3-1* , we can measure gene expression changes of inferred target genes. Protoplasts isolated from unifoliate leaves were transfected, incubated overnight, and harvested at the Zeitgeber time points ZT4, ZT8, ZT12, ZT16, ZT20, and ZT24. Cell viability and GFP expression were examined prior to harvest. Approximately 90% of protoplasts were viable and about 70% of transfected protoplasts were expressing GFP signals at all time points, while non-transfected protoplasts showed no fluorescence **(Figures B-C)** . Our RT-qPCR analysis confirmed that *GmELF3-1* was expressed significantly higher in transfected protoplasts than in non-transfected control protoplasts by a range of 150,000-fold to 420,000-fold across the 6 time points **(Figure E)** . *GmELF3-1* overexpression affected mRNA expression levels and patterns of the inferred target genes. *GmPRR7-1* was upregulated at ZT16 and ZT20 by 7-fold and 4-fold, respectively, in transfected protoplasts than in non-transfected control protoplasts **(Figure F)** . *GmPRR7-2* was upregulated at ZT16 and ZT20 by 5-fold and 2-fold, respectively, while it was downregulated at ZT24 by 1.5-fold **(Figure G)** . Significant upregulation of *GmCOL1a* was observed at ZT4 by 3,000-fold, while upregulation at other time points was marginable **(Figure H)** .

In the second approach, we examined the effects of *GmELF3-1* knockout using CRISPR-Cas9 targeted mutagenesis as a counterpart of the overexpression approach. Protoplasts were transfected with the following ribonucleoprotein (RNP) complexes ( **Tables 3 and 4)** : single guide RNA (sgRNA) on the positive strand (g+) bound to Cas9 nuclease, RNP(g+); sgRNA on the negative strand (g-) bound to Cas9 nuclease, RNP(g-); or paired guide RNA (g+/-) bound to Cas9 nickase, RNP(g+/-). Protoplasts were harvested after over 48 hours incubation at the Zeitgeber time points ZT12 and ZT20. Approximately 60% of protoplasts were viable at the time of harvest based on FDA staining **(Figure D)** . RT-qPCR analysis showed that *GmELF3-1* expression levels were moderately upregulated at ZT12: with RNP(g+) by 3-fold, with RNP(g-) by 1.5-fold, and with RNP(g+/-) by 2.5-fold compared with the negative control **(Figure I)** . This observation was puzzling because we expected our CRISPR experiments would induce a single nucleotide mutation or a small indel that would have little or no effects on *GmELF3-1* mRNA abundance. However, it is possible that mutagenized *GmELF3-1* may affect expression of its target gene(s) controlling *GmELF3-1* , resulting in the observed upregulation of *GmELF3-1* itself when *GmELF3-1* normally is not expressed. Expression levels of the inferred target genes were observed in these CRISPR-treated protoplasts **(Figures J-L)** . *GmPRR7-1* was significantly upregulated at ZT12: with RNP(g+) by 3-fold, with RNP(g-) by 5-fold, and with RNP(g+/-) by 7-fold compared with the negative control **(Figure J)** , while *GmPRR7-2* showed varying expression levels at ZT12: downregulation with RNP(g+) by 1.2-fold, upregulation with RNP(g-) by 1.6-fold, and downregulation with RNP(g+/-) by 1.1-fold **(Figure K)** . *GmCOL1b* was significantly upregulated at ZT12 with RNP(g+) by 2.7-fold, RNP(g-) by 1.3-fold, and RNP(g+/-) by 2.1-fold **(Figure L)** . In the evening at ZT20, *GmELF3-1* expression levels were generally maintained with minor down- or up-regulation: with RNP(g-) by 0.9-fold and RNP(g+/-) by 1.6-fold, respectively **(Figure M)** . *GmPRR7-1* was moderately downregulated with RNP(g+) by 0.8-fold, RNP(g-) by 0.9-fold, and RNP(g+/-) by 0.9-fold at ZT20 **(Figure N)** . Similarly, *GmPRR7-2* showed downregulation by 1.4-fold with RNP(g+), RNP(g-), and RNP(g+/-) **(Figure O)** . On the other hand, *GmCOL1a* showed significant upregulation with RNP(g+) by 7-fold, RNP(g-) by 6-fold, and RNP(g+/-) by 2-fold at ZT20 **(Figure P).**

These observations demonstrate critical regulatory interactions of GmELF3-1 with *GmPRR7-1, GmPRR7-2,* and *GmCOL1a* at varying times of the day. Two major implications of our results are a feedback regulation of *PRR* genes by the EC is likely conserved in the circadian clock of soybean, and that the EC controls *GmCOL1* expression directly or indirectly. Limitations of this study include minimal sampling time points of the CRISPR experiment. Soybean’s circadian clock and flowering genes controlled by the clock exhibit daily oscillation patterns and their expression levels change quickly, thus using 2 time points may not capture an accurate regulation of rhythmic gene expression. In addition, experimentally determining whether a transcription factor/regulator is a transcriptional activator or a repressor is a difficult problem, especially when actions of a transcription factor change quickly within a short period of time. We cannot rule out a possible indirect regulation through multiple feedback loops within the circadian clock gene network that may mislead interpretations of gene regulatory interactions in our study. Moreover, our CRISPR approaches will require further verification for successful mutagenesis and for induced loss-of-function of GmELF3-1. Regardless, this work aids in characterizing the roles of GmELF3-1 and provides a framework in network inference and experimental verification of gene regulatory networks.

## Methods

**Plant Growth Condition and Sampling**

The *Glycine max* accession PI 518671 (Williams 82) cultivar seeds were grown in Sunshine Mix #4 Professional Growing Mix with Mycorrhizae and Vermiculite in a 6:1 respective ratio. Plants were grown in a controlled growth chamber under long day conditions (LD, 14h light/10h dark) at 30°C/28°C. Soil conditions were monitored daily, establishing moisture was maintained between 40-50% and pH at 7. Soybean seedlings 4-5 days post-germination that had fully expanded unifoliate leaves were used for further analysis.

**Plasmid Construction**

The *GmELF3-1* cDNA was cloned and subcloned into the p2GFW7 vector harboring the *GFP* gene, driven by the 35S promoter (VIB-UGENT Center for Plant Systems Biology; https://gatewayvectors.vib.be/collection/p2fgw7 ) as described previously (Alcantara et al., 2022) . Further information about the p2GFW7-ELF3-1 plasmid can be inquired by contacting the corresponding author Yoshie Hanzawa (yoshie.hanzawa@csun.edu).

**RNP Assembly**

This study used two gRNA, designed on Benchling ( https://www.benchling.com/ ), and used as follows: single guide RNA (sgRNA) on the forward strand of Exon 1 of *GmELF3-1,* sgRNA on the reverse strand of Exon 1 of *GmELF3-1,* and paired gRNAs on the forward and reverse strands of Exon 1 of *GmELF3-1* spaced 20 nucleotides apart. Designed gRNAs were ordered from IDT (https://www.idtdna.com/) to be synthesized as sgRNAs. The RNP complex was produced by mixing the appropriate *S.p.* Cas9 enzyme (Cas9-nuclease for sgRNA and Cas9-nickase for paired gRNAs) with one or more sgRNAs in an equimolar amount (1:1 ratio) in Cas9 Dilution Buffer (30 mM HEPES, 150mM KCl, pH 7.5). The mixture was incubated for 10 minutes at room temperature for RNP complex formation and used for protoplast delivery in vivo.

**Protoplast Isolation**

The protoplast isolation followed the procedures described previously (Wu and Hanzawa, 2018; Alcantara et al., 2022) with minor modifications. Soybean mesophyll cells from unifoliate leaves (7d after sowing) were separated by the leaf-tape method (Wu et al. 2009; Alcantara et al., 2022) . The cells were incubated in 10mL of an enzymatic digestion solution (0.02M MES pH 5.7, 1.5% w/v Cellulase R-10, 0.50% w/v Macerozyme, 0.20% w/v Pectolyase Y-23, 0.1M CaCl _2_ , 0.1% BSA (7.5% stock), and 0.4M w/v D-Mannitol) under low light at 22 ^º^ C with gentle agitation of 100rpm. Digestion was quenched by the addition of W5 (154 mM NaCl, 125 mM CaCl _2_ , 5 mM KCl, 2 mM MES pH 5.7) and filtered by a 70mm nylon mesh. Protoplast cells were resuspended in prechilled W5 to a final concentration of 2 x 10 ^5^ mL ^-1^ , predetermined by hemocytometer. Succeeding an ice bath incubation for 30 minutes, protoplasts were resuspended in MMg (4 mM MES pH 5.7, 400 mM D-Mannitol, 15 mM MgCl _2_ ) to a final concentration of 2 x 10 ^5^ mL ^-1^ .

**Protoplast Transfection for Overexpression**

For overexpression transfection assay, 20 μg of pGFW7-ELF3-1 plasmid was added to 10 ^5^ mL ^-1^ protoplasts, mediated by an equal volume of freshly prepared PEG solution (20% w/v PEG4000, 400 mM D-Mannitol, 100 mM CaCl _2_ ) and immediately mixed by inversion. The mixture was incubated for 12 minutes at room temperature, after which, 8 mL of W5 was slowly added to quench transfection. Pellets were collected after 100 x g centrifugation for 5 minutes and resuspended in WI solution (4 mM MES pH 5.7, 500 mM Mannitol, 20 mM KCl) to a final concentration of 10 ^5^ mL ^-1^ . A 6-well tissue culture plate was coated with 50% v/v sterile calf serum where 1 mL of transfected protoplasts were transferred and incubated at 22 ^º^ C overnight in the dark. At roughly 24 hours post transfection, a sample of protoplast cells were stained with fluoresceine diacetate (FDA) and imaged under *Leica* confocal microscope. GFP signals were verified by confocal laser microscopy at the time of harvest.

**Protoplast Transfection for Targeted Mutagenesis**

For *in vivo* CRISPR/Cas9 transfection assay, 25 μL RNP complex (1 mM sgRNA: 1 mM Cas9) was added to 1 mL aliquot of 10 ^5^ mL ^-1^ protoplasts, mediated by an equal volume of freshly prepared PEG solution, and immediately mixed by inversion. The transfection mixture was incubated for 30 minutes in the dark at room temperature, after which, 8 mL of W5 was slowly added to quench transfection. Pellets were then collected after 100 x g centrifugation for 5 minutes and resuspended in WI solution to a final concentration of 10 ^5^ mL ^-1^ . Transfected cells were transferred to a 50% v/v sterile calf serum pre-coated tissue culture plate and incubated at 25 ^º^ C for 48 hours in the dark. At roughly 48 hours post transfection, a sample of protoplast cells were stained with FDA and imaged under confocal microscopy.

**RNA Extraction, cDNA Synthesis, and Quantitative RT-PCR**

Total RNA was extracted from harvested protoplast cells and invasive genomic DNA removed with DNAse using the Invitrogen RNA kit (Invitrogen, CA, USA), per the manufacturer’s protocol. First-strand cDNA was synthesized using the Reverse Transcriptase Kit, per the manufacturer’s protocol and diluted to 1:20 for RT-qPCR application. RT-qPCR was performed using the QuantStudio3 with samples containing the fluorogenic probe SYBR™ Green. The following amplification settings were performed: 2-minute hold at 50°C, 10 minutes at 95°C, a PCR of 15 seconds at 95°C, and ending with 1 minute at 60°C where amplification was captured. These settings were set to repeat for 50 cycles. The Ct values were then analyzed on Thermo Fisher server (https://apps.thermofisher.com/). Relative gene expression was normalized against the housekeeping gene *GmPBB2* and calculated as 2 ^-DCt^ as described in Wu et al 2014 and Livak and Schmittgen 2001 (Livak & Schmittgen, 2001; Wu et al., 2014) .

## Reagents

**Table:** 

**Accession**	**ID**	**Available From**
Williams 82	PI 518671	USDA

**Table 1.** Soybean accession.

**Table:** 

**Plasmid**	**Gene ID**
p2FGW7-ELF3-1	Glyma.04G050200

**Table 2.** Fusion construct of *GmELF3-1* and Gene ID.

**Table:** 

**gRNA Name**	**gRNA sequence (5’ – 3’)**	**Site**	**Target Gene Name**
GmELF31-F	GAGGCCCAAGAGCACCACCT	850	*GmELF3-1* +
GmELF3-1-R	ACATGTAGTCTAGGGAACAT	799	*GmELF3-1* -

**Table 3.** Guide RNA sequences and targeted cut site.

**Table:** 

**Cas Endonuclease**	**Size**	**Bacterial Source**	**Pam Recognition Site**	**Reference**
SpCas9 nuclease	4.1 kb	*Streptococcus pyogenes*	3’ NGG	(Kleinstiver et al., 2015)
SpCas9 nickase	4.1 kb	*Streptococcus pyogenes*	Enhanced specificity, 3’ NGG	(Mali et al., 2013)

**Table 4.** Cas endonucleases and their respective recognition site.

**Table:** 

**Primer Name**	**Primer sequence (5’ – 3’)**	**Target Gene Name**	**Gene ID**
GmPBB2-F GmPBB2-R	TGCCGAAGAAACGCAATGCTTCAA TGCAGCAAGTGAACCTGATCCCAT	*GmPBB2*	Glyma.14G01850
GmELF3-1­-F GmELF3-1­-R	TGTTCTGCCACTCAACCCAA TGATTGGCGTGAGTTACATT	*GmELF3-1*	Glyma.04G050200
GmPRR7-1-F GmPRR7-1-R	TATGAAGTTATTGAAGCAGC AGAATCATGAGATGACATCA	*GmPRR7-1*	Glyma.10G048100
GmPRR7-2-F GmPRR7-2-R	GTCTGCTTTCTCAAGGTACA GGAGGATTGCCGCTAGAATG	*GmPRR7-2*	Glyma.13G135900
GmCOL1a-F GmCOL1a-R	CGCCTCGCTGACGTGGCACG TTGTCGTTGTTGCCGGGGGC	*GmCOL1a*	Glyma.08G255200

**Table 5.** RT-qPCR primers targeting CDS sequences of selected targeted genes using the obtained sequences from https://phytozome-next.jgi.doe.gov/ in *Williams82.a2.v1* .

## References

[R1] Alabadí David, Yanovsky Marcelo J., Más Paloma, Harmer Stacey L., Kay Steve A. (2002). Critical Role for CCA1 and LHY in Maintaining Circadian Rhythmicity in Arabidopsis. Current Biology.

[R2] Alcantara Michelle, Acosta Patrick, Azatian Ara , Calderon Carlos , Candray Kevin , Castillo Natalie, Coria-Gomez Luis , Duran Jose , Fam Justina, Hernandez-Segura Diego , Hidalgo Lennix , Huerta Carlos , Jordan Shane , Kagan Kimberly , Loya Karla, Martinez Eduardo, Musaev Kirill , Navarro Roxana, Nazarians Narek , Paglia Robert , Robles Gabriela, Simmons Taylor , Smith Shawn, Soudani Faisel , Valenzuela Emily, Villalobos Jessica , Iftikhar Hira, Hanzawa Yoshie (2022). Experimental Verification of Inferred Regulatory Interactions of EARLY FLOWERING 3 (GmELF3-1) in Glycine max.

[R3] Bu Tiantian, Lu Sijia, Wang Kai, Dong Lidong, Li Shilin, Xie Qiguang, Xu Xiaodong, Cheng Qun, Chen Liyu, Fang Chao, Li Haiyang, Liu Baohui, Weller James L., Kong Fanjiang (2021). A critical role of the soybean evening complex in the control of photoperiod sensitivity and adaptation. Proceedings of the National Academy of Sciences.

[R4] Chow Brenda Y., Helfer Anne, Nusinow Dmitri A., Kay Steve A. (2012). ELF3 recruitment to the
*PRR9*
promoter requires other Evening Complex members in the Arabidopsis circadian clock. Plant Signaling & Behavior.

[R5] Creux Nicky, Harmer Stacey (2019). Circadian Rhythms in Plants. Cold Spring Harbor Perspectives in Biology.

[R6] Ezer Daphne, Jung Jae-Hoon, Lan Hui, Biswas Surojit, Gregoire Laura, Box Mathew S., Charoensawan Varodom, Cortijo Sandra, Lai Xuelei, Stöckle Dorothee, Zubieta Chloe, Jaeger Katja E., Wigge Philip A. (2017). The evening complex coordinates environmental and endogenous signals in Arabidopsis. Nature Plants.

[R7] Harmon Frank G., Imaizumi Takato, Kay Steve A. (2018). The Plant Circadian Clock: Review of a Clockwork
*Arabidopsis*. Annual Plant Reviews online.

[R8] Huang He, Nusinow Dmitri A. (2016). Into the Evening: Complex Interactions in the Arabidopsis Circadian Clock. Trends in Genetics.

[R9] Kleinstiver Benjamin P., Prew Michelle S., Tsai Shengdar Q., Topkar Ved V., Nguyen Nhu T., Zheng Zongli, Gonzales Andrew P. W., Li Zhuyun, Peterson Randall T., Yeh Jing-Ruey Joanna, Aryee Martin J., Joung J. Keith (2015). Engineered CRISPR-Cas9 nucleases with altered PAM specificities. Nature.

[R10] Li Cong, Li Ying-hui, Li Yanfei, Lu Hongfeng, Hong Huilong, Tian Yu, Li Hongyu, Zhao Tao, Zhou Xiaowei, Liu Jun, Zhou Xinan, Jackson Scott A., Liu Bin, Qiu Li-juan (2020). A Domestication-Associated Gene GmPRR3b Regulates the Circadian Clock and Flowering Time in Soybean. Molecular Plant.

[R11] Livak Kenneth J., Schmittgen Thomas D. (2001). Analysis of Relative Gene Expression Data Using Real-Time Quantitative PCR and the 2−ΔΔCT Method. Methods.

[R12] Lu Sijia, Dong Lidong, Fang Chao, Liu Shulin, Kong Lingping, Cheng Qun, Chen Liyu, Su Tong, Nan Haiyang, Zhang Dan, Zhang Lei, Wang Zhijuan, Yang Yongqing, Yu Deyue, Liu Xiaolei, Yang Qingyong, Lin Xiaoya, Tang Yang, Zhao Xiaohui, Yang Xinquan, Tian Changen, Xie Qiguang, Li Xia, Yuan Xiaohui, Tian Zhixi, Liu Baohui, Weller James L., Kong Fanjiang (2020). Stepwise selection on homeologous PRR genes controlling flowering and maturity during soybean domestication. Nature Genetics.

[R13] Lu Sijia, Zhao Xiaohui, Hu Yilong, Liu Shulin, Nan Haiyang, Li Xiaoming, Fang Chao, Cao Dong, Shi Xinyi, Kong Lingping, Su Tong, Zhang Fengge, Li Shichen, Wang Zheng, Yuan Xiaohui, Cober Elroy R, Weller James L, Liu Baohui, Hou Xingliang, Tian Zhixi, Kong Fanjiang (2017). Natural variation at the soybean J locus improves adaptation to the tropics and enhances yield. Nature Genetics.

[R14] Mali Prashant, Aach John, Stranges P Benjamin, Esvelt Kevin M, Moosburner Mark, Kosuri Sriram, Yang Luhan, Church George M (2013). CAS9 transcriptional activators for target specificity screening and paired nickases for cooperative genome engineering. Nature Biotechnology.

[R15] McClung C. (2019). The Plant Circadian Oscillator. Biology.

[R16] Mizoguchi Tsuyoshi, Wheatley Kay, Hanzawa Yoshie, Wright Louisa, Mizoguchi Mutsuko, Song Hae-Ryong, Carré Isabelle A., Coupland George (2002). LHY and CCA1 Are Partially Redundant Genes Required to Maintain Circadian Rhythms in Arabidopsis. Developmental Cell.

[R17] Mizuno Takeshi, Nomoto Yuji, Oka Haruka, Kitayama Miki, Takeuchi Aya, Tsubouchi Mayuka, Yamashino Takafumi (2014). Ambient Temperature Signal Feeds into the Circadian Clock Transcriptional Circuitry Through the EC Night-Time Repressor in Arabidopsis thaliana. Plant and Cell Physiology.

[R18] Nakamichi, N., Kiba, T., Henriques, R., Mizuno, T., Chua, N. H., Sakakibara, H. (2010). PSEUDO-RESPONSE REGULATORS 9, 7, and 5 are transcriptional repressors in the Arabidopsis circadian clock. *Plant Cell, 22* (3):594-605. doi: 10.1105/tpc.109.072892. 10.1105/tpc.109.072892 PMC2861452 20233950

[R19] Nakamichi, N., Kiba, T., Kamioka, M., Suzuki, T., Yamashino, T., Higashiyama, T., Sakakibara, H., Mizuno, T. (2012). Transcriptional repressor PRR5 directly regulates clock-output pathways. *Proc Natl Acad Sci U S A, 109* (42):17123-8. doi: 10.1073/pnas.1205156109. 10.1073/pnas.1205156109 PMC3479524 23027938

[R20] Ronald J, Davis SJ (2017). Making the clock tick: the transcriptional landscape of the plant circadian clock.. F1000Res.

[R21] Wu Faqiang, Kang Xiaohan, Wang Minglei, Haider Waseem, Price William B., Hajek Bruce, Hanzawa Yoshie (2019). Transcriptome-Enabled Network Inference Revealed the GmCOL1 Feed-Forward Loop and Its Roles in Photoperiodic Flowering of Soybean. Frontiers in Plant Science.

[R22] Wu Faqiang, Price Brian William, Haider Waseem, Seufferheld Gabriela, Nelson Randall, Hanzawa Yoshie (2014). Functional and Evolutionary Characterization of the CONSTANS Gene Family in Short-Day Photoperiodic Flowering in Soybean. PLoS ONE.

[R23] Wu FH, Shen SC, Lee LY, Lee SH, Chan MT, Lin CS (2009). Tape-Arabidopsis Sandwich - a simpler Arabidopsis protoplast isolation method.. Plant Methods.

[R24] Xia Zhengjun, Watanabe Satoshi, Yamada Tetsuya, Tsubokura Yasutaka, Nakashima Hiroko, Zhai Hong, Anai Toyoaki, Sato Shusei, Yamazaki Toshimasa, Lü Shixiang, Wu Hongyan, Tabata Satoshi, Harada Kyuya (2012). Positional cloning and characterization reveal the molecular basis for soybean maturity locus
*E1*
that regulates photoperiodic flowering. Proceedings of the National Academy of Sciences.

[R25] Yue Yanlei, Liu Nianxi, Jiang Bingjun, Li Mu, Wang Haijie, Jiang Ze, Pan Huanting, Xia Qiuju, Ma Qibin, Han Tianfu, Nian Hai (2017). A Single Nucleotide Deletion in J Encoding GmELF3 Confers Long Juvenility and Is Associated with Adaption of Tropic Soybean. Molecular Plant.

